# Valence ratings of recorded media for adults with and without hearing loss

**DOI:** 10.1080/14992027.2026.2710964

**Published:** 2026-08-05

**Authors:** Haiping Huang, Anne-Charlotte J. Belloeil, Todd A. Ricketts, Javier Santos, Rebecca Krivoshey, Gabrielle H. Buono, Erin M. Picou

**Affiliations:** a Department of Hearing and Speech Sciences, Vanderbilt University, Nashville, TN, USA; b Department of Psychology and Human Development, Vanderbilt University, Nashville, TN, USA; c Department of Hearing and Speech Sciences, Vanderbilt University Medical Center, Nashville, TN, USA; d Departamento de Otorrinolaringología, Universidad de Navarra, Navarra, Spain; e Department of Otolaryngology, Thomas Jefferson University, Philadelphia, PA, USA; f United States Air Force, San Antonio, TX, USA

**Keywords:** Hearing loss, emotion, television, valence, background noise

## Abstract

**Objectives:**

This study evaluated valence ratings in response to recorded media under a variety of listening situations.

**Design and Study Sample:**

Younger adults with normal hearing (*n* = 20), older adults with typical hearing (*n* = 20), and older adults with hearing loss (*n* = 20) provided ratings of valence (the degree to which they felt pleasant) for audio-only (Experiment 1) and audiovisual (Experiment 2) recorded media clips. Clips varied by dialogue-to-background ratio and loudspeaker configuration (television, stereo, surround sound).

**Results:**

In both experiments, older listeners with hearing loss provided significantly lower valence ratings than their typical hearing peers, but only when the surround sound loudspeakers were used or when the dialogue-to-background ratio was +7 dB. Older listeners provided lower valence ratings when the loudspeaker was either stereo or surround sound or when dialogue-to-background ratio was +12 dB, but only when visual cues were not available.

**Conclusion:**

Older adults with hearing loss exhibit disrupted ratings of valence to recorded media. The effect could be mitigated with improved dialogue-to-background ratios of television shows and movies, but not with surround sound systems.

## Introduction

Watching recorded media (e.g. television, video) is an important component of modern life for most adults. It is estimated that more than 60% of the adults 20 years and older in the United States spend at least 2 hours a day watching recorded media (Yang et al. 2019). Further, adults aged 65 years and older spend threefold more waking time watching television than younger adults; yet, they report less enjoyment in watching television compared to their younger peers (Depp et al. 2010). One of the motivations for people to watch television is for emotion and mood improvement (Barwise, Bellman, and Beal 2020). Therefore, it is possible that these older adults enjoyed watching television less because the activity did not elevate their emotion and mood.

### Emotional responses

In this work, we operationally define emotional response as the degree to which someone is affected by a stimulus. An emotional response is quick (1–2 sec), directed towards an object or event, and is felt and appraised (Mulligan and Scherer 2012). This is in contrast to other types of affective phenomena, such as moods (which are slower and event specific) or temperament (Davidson, Sherer, and Goldsmith 2009). The dimensional view of emotion (circumplex model; Russell 1980) provides a convenient framework for measuring the extent to which an individual is affected by stimuli by having them rate their response to the stimulus along a combination of two or more dimensions. Under this framework, most of the variability in the judgments of emotional stimuli can be accounted for with three dimensions: arousal (exciting vs. calming), valence (pleasant vs. unpleasant), and dominance (dominant vs. submissive; Bradley and Lang 2007; Russell and Mehrabian 1977). Of these dimensions, the combination of the first two dimensions (arousal and valence) accounts for most of the variability in emotion (Bradley and Lang 1994).

Consistent with the view that pleasant or appetitive stimuli serve to enhance a person’s well-being (Lang 1995), pleasant stimuli have been shown to aid stress recovery (e.g. nature sounds; Annerstedt et al. 2013). Therefore, it is possible that older adults report less enjoyment watching television than their younger peers (Depp et al. 2010) could be investigated by looking at how they are responding emotionally to media content.

### Effects of hearing loss

Hearing loss is a prevalent health condition among our older listeners (Lin et al. 2011). Past studies using nonspeech sounds or speech sounds have each shown emotion perception deficits among listeners with hearing loss. For example, Picou (2016) found that, in response to non-speech sounds, older listeners with hearing loss rated pleasant sounds as less pleasant and unpleasant sounds as less unpleasant, compared to their peers with typical hearing. Similarly, Christensen et al. (2019) showed in a speech emotion recognition task that more hearing loss was associated with both slower response time and reduced accuracy in an emotion recognition task. Studies of emotion perception are often conducted in laboratory settings.

Recently, Holman and Naylor (2025) used ecological momentary assessment (EMA) to measure emotions in adults with and without hearing loss over a 10-day-period. The transitory emotional response was modelled dimensionally using the valence and arousal ratings. Collapsed across conversational and non-conversational listening situations, decreased valence was associated with increased hearing handicap among older listeners. Notably, TV watching was one frequently reported scenario among respondents, constituting around 30–40% of total activities. The study provides important guidance: there are indeed group differences in emotion perception in day-to-day listening, and that TV watching may be an especially relevant listening situation to investigate.

General recorded media contents (television shows and movies) are unique from affective stimuli used in past studies in several important ways. First, they frequently involve a mix of speech and nonspeech sounds. Decoding the emotion in speech likely requires analysis of both prosodic and semantic cues (Richter and Chatterjee 2021), while acoustic features such as spectrotemporal modulation cue for valence responses to nonspeech sounds (Huang, Picou, and Martens 2026). Further, the background sounds are often relevant to the running plot (Kozloff 2000), rather than simply masking speech. How listeners emotionally respond to this complex mixture is not yet fully understood.

### Effects of age, loudspeaker configuration, and dialogue-to-background ratios

Another factor affecting how older listeners emotionally respond to recorded media may be ageing. The effects of ageing on recognition of emotion in others demonstrates older adults have lower recognition performance than younger adults (e.g. Christensen et al. 2019). Yet, the effects of ageing on emotional response to sounds have not been conclusive. Some showed that age did not have an effect on emotional response to videos of real-world events (Pavic et al. 2024), or nonspeech sounds (Picou 2016). Conversely, a study using film as a stimulus showed that older listeners perceived certain negative emotion types, such as disgust and fear as more negative than do younger peers (Fernandez-Aguilar et al., 2018). Other than apparent stimulus-related differences, it is uncertain what contributed to the discrepancies across these studies.

Specific to television viewing, but not considered in the past studies on emotion perception (e.g. Christensen et al. 2019; Picou 2016), are loudspeaker configuration and dialogue-to-background ratio. Physical loudspeaker components may affect listening experiences (e.g. Holman 2012). In a recent survey study investigating the television listening experience among people with and without hearing loss, about one-third of the respondents reported the use of external loudspeakers (Strelcyk and Singh 2018). Among these listeners who specified their audio equipment set-up, one-third reported using at least two external loudspeakers, demonstrating that a variety of loudspeaker configurations are common.

Lastly, types and levels of background sounds vary across television programs, often due to the purpose of the content (Holman 2012). When dialogue comprehension is of importance and there is excessive background sound, this often leads to complaints from the audience (Armstrong 2016). Therefore, we were interested in examining whether the effects of hearing loss and age on emotional response depends on the relative levels of dialogue and non-dialogue sounds, that is, the dialogue-to-background ratio.

### Purpose

The purpose of this project was to evaluate ratings of valence in response to auditory-only (Experiment 1) and auditory-visual (Experiment 2) recorded media for adults with and without hearing loss. Because recorded media experiences inherently vary also based on the loudspeakers used and on the dialogue-to-background ratio, a variety of realistic loudspeaker configurations and dialogue-to-background ratios were used.

Prior studies showed emotion perception deficits associated with hearing loss (Christensen et al. 2019; Holman and Naylor 2025; Picou 2016). Therefore, we expected that listeners with hearing loss would rate recorded media clips as less pleasant than their peers with typical hearing. It was also expected that listeners who were younger and older would respond similarly to each other, because age has not been related to ratings of valence previously (Picou 2016).

Based on the general perception of multi-loudspeaker-induced enhancement on envelopment or spaciousness for entertainment purposes (Moiragias and Mourjopoulos 2023), we expected stereo and surround sound system to alleviate negative effects of hearing loss. Two different dialogue-to-background ratios were used to evaluate whether the effects of hearing loss depended on dialogue audibility in the recorded stimuli. Based on the consistent findings that adults with hearing loss require a better signal-to-noise ratio to understand speech in noise than their peers with normal hearing (e.g. Dubno, Dirks, and Morgan 1984), we expect this effect carries over to ratings of valence. In other words, we expected the effects of hearing loss to depend on the dialogue-to-background ratio.

## Experiment 1 (auditory only): materials and methods

### Participants

**Figure 1.**
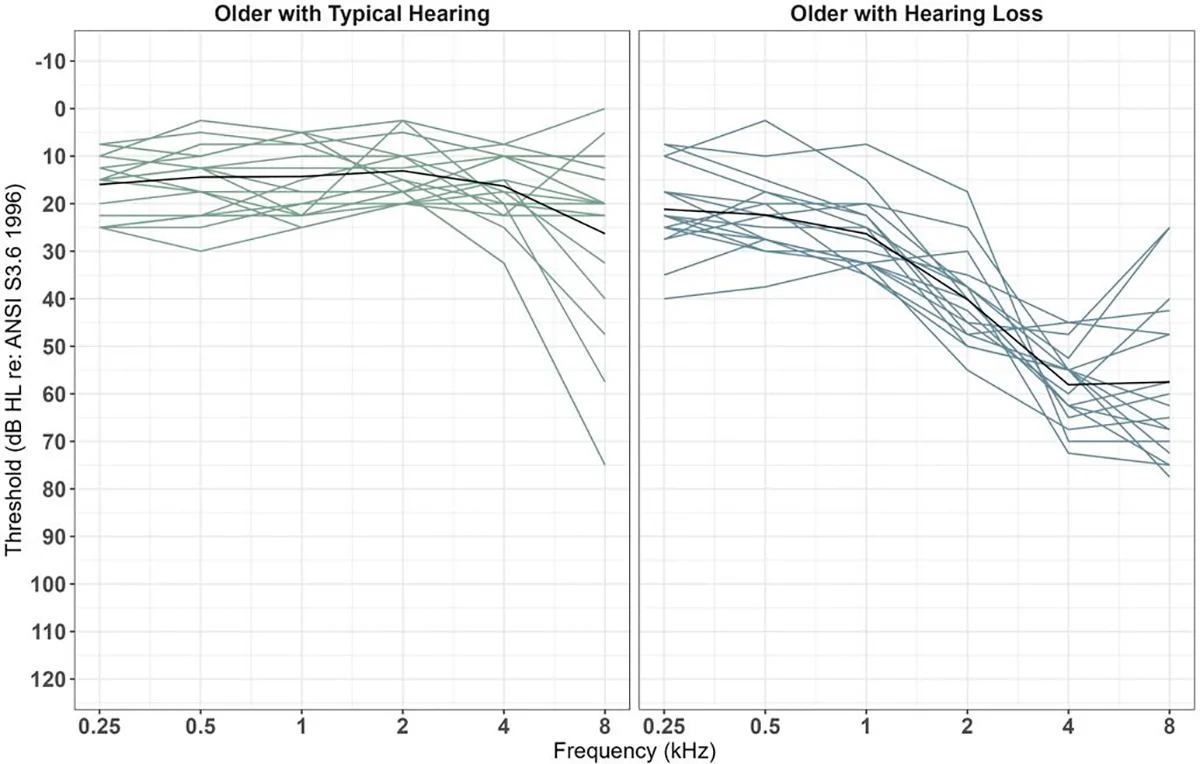
Individual (colours) and mean (black lines) better ear, audiometric thresholds as a function of frequency for older participants with typical hearing (left panel) and older participants with hearing loss (right panel). Younger adults with normal hearing passed a hearing screening at 25 dB HL (not pictured).

A total of 60 adults in three groups (each *n* = 20) participated in this study. The first two groups included younger (aged 18–35 yrs, M = 24.4 yrs) and older (aged 50–79 yrs, M = 69.8 yrs) listeners with typical hearing. For the younger group, this was defined as frequency-specific, air-conduction thresholds of no worse than 25 dB HL from 0.5 through 8 kHz. For the older group, this was defined as normal air-conduction thresholds at 0.5–3 kHz and no worse than mild to moderate hearing loss ≥ 4 kHz (International Organization for Standardization 2017). The third group included older adults (ages 50–81 yrs, M = 70.2 yrs) with mild sloping to moderately-severe air-conduction, hearing thresholds (see Figure 1 for better ear thresholds for two groups of older listeners). Maximum better ear hearing thresholds through 4 kHz were limited to no worse than 65 dB HL in this group during recruitment and only participants who denied experience with hearing aids were included. Participants with hearing loss had normal middle ear pressure and compliance and air-bone gaps of less than 15 dB. All participants had symmetrical hearing based on no difference in pure-tone average larger than 15 dB HL (averaged across 0.5, 1, 2, and 3 kHz; Steiger 2005). Participants had normal or corrected normal vision by self-report. All participants self-reported to be native speakers of American English. Study procedures were approved by Vanderbilt University’s Institutional Review Board (#161938). Participants were paid for their time on a per session basis. The same participants completed study procedures in the current study and in a companion study (Ricketts et al. 2026).

### Test materials

The stimulus selection and manipulation are detailed in Appendix A. In brief, our stimulus selection aimed to include a variety of materials because a listener’s experience depends on a multitude of factors (material, background sound, emotional response; for example, Moiragias and Mourjopoulos 2023). The current work selected materials with realism from commercial television shows and movies. We screened approximately 100 television shows and movie clips for possible inclusion. At the end of screening, a total of 12, 60-second clips (6 audio and 6 audiovisual) were digitised to form the final test materials. The 6 clips allowed for testing in 6 conditions, specifically three loudspeaker configurations and two dialogue-to-background ratios (described below). The clips were then edited (Adobe Audition, Version 2017.1.1, Build 10.1.1.11) to manipulate presentation level and dialogue-to-background ratio *within* all clips while maintaining the natural sound characteristics as much as possible. The relative level of the dialogue in the centre channel to the background in the other channels was manipulated to be either +7 or +12 dB. These ratios were commonly observed in the media clips screened for potential study inclusion. The total range of dialogue-to-background ratios observed during screening was 0 to +30 dB.

To collect listeners’ emotional response, we asked participants to provide valence and arousal ratings using the Self-Assessment Manikin (SAM; Bradley and Lang 1994). The SAM is a non-verbal, picture-oriented instrument for measuring emotion. It includes three domains: valence, arousal, and dominance. Each has 5 cartoon figures along with a range of 1 to 9 scale underneath, equally spaced, representing the range of emotions along the relevant domain (e.g. smiling to frowning). Participants were instructed to provide ratings based on the numeric scale. For the current study, we only used the valence and arousal domains. We included the captions “how pleasant/unpleasant do you feel” and “how excited or calm do you feel” above the cartoon figures for valence and arousal, respectively. Because ratings of arousal were not responsive to hearing loss or sound filtering in our past works, results and brief discussion of arousal are reported in Appendix B.

### Test conditions

The dialogue-to-background ratios were +7 and +12 dB. The three loudspeaker configurations were: 1) “television” - embedded stereo television speakers, (2) “stereo” - external stereo speakers, and (3) “surround sound” - a five-channel surround sound speaker system. The final calibrated five-channel test materials were down-mixed to stereo versions using the Adobe Audition in the stereo condition. Prior to testing, the presentation levels of the centre dialogue were calibrated to 70 dBA level for each loudspeaker configuration using a steady-state noise with the same long-term average spectral shape and level as the concatenated speech materials.

### Test environment

All participants were seated in the centre of a double-walled, sound-attenuating audiometric test booth. The television and loudspeakers were placed approximately 1.1 m from the centre of the seat position. The television that delivered the audio signal was placed on a wooden shelf directly in front of the participants. The television was covered with blackout felt curtain to obscure the visual components.

## Television loudspeaker configuration

Two down-firing speakers on a television (Sharp LC-32LB481U, 32-inch) were activated. All advanced audio processing features within the television were disabled (i.e. virtual surround sound) and sound delivery was set to standard stereo.

## Stereo loudspeaker configuration

Two loudspeakers (Tannoy Precision 6 P) placed at ± 30 degrees were used for the external stereo speaker conditions.

## Surround sound loudspeaker configuration

Consistent with the recommended 5.1. surround sound configuration (ITU-R BS.775), four loudspeakers (Definitive Technologies Model BP-2X) were placed at ± 30 and ± 110 degree angles relative to the listener. The centre channel speaker (Tannoy Precision 6 P) was placed directly in front of participants, directly below the television.

The test materials were delivered using a laptop computer (Dell) and either five- or two- channels of an Echo Firewire12 audio interface. Audio materials were output from the audio interface to the television via HDMI or to the direct audio input (pass-through) of a Yamaha Natural Sound AV Receiver (RX-V463) for stereo and surround sound loudspeaker configurations.

### Procedures

Prior to data collection, participants provided informed consent and underwent hearing evaluation using pure-tone air conduction audiometry and immittance measures. During data collection, the six, 60-second audio-only clips were presented in random order. Each clip was divided into six, 10-second segments. For each segment, a participant listened to one of the 6 conditions (3 loudspeaker configurations × 2 dialogue-to-background ratios). The order of conditions within a clip was random without replacement. In other words, participants rated the six different combinations of conditions (3 speaker configurations × 2 dialogue-to-background ratios) once per 60-second clip and completed the ratings 6 times for a total of 36 ratings (6 conditions × 6 clips). After each 10-second segment, participants completed the SAM scale on a computer tablet regarding valence and arousal. The instruction to the participants was “You will hear short segments of a television show or a movie. Please listen to each clip carefully. As you’re listening, think about how the clip made you feel. You will be asked to rate how pleasant or unpleasant the clip made you feel and also how excited or calm it made you feel ... . You will listen to 6 clips in a row. They are all from the same show or movie.” They also answered questions about speech intelligibility and sound quality in the same set of questionnaire; these data are presented elsewhere (Ricketts et al. 2026).

### Data analysis

We used a multilevel path-analysis framework to conduct mean contrasts using Mplus version 8.8 (Muthén and Muthen 2017). The outcome variable was valence and the predictor variables included level-1 variables (within-participant) of dialogue-to-background ratios and speaker configuration and a level-2 variable (between-participant) of group (younger normal hearing, older with typical hearing, older with hearing loss). Each condition included six ratings for each participant. We used multilevel path analysis framework to accommodate the nesting of trials within persons (Silva, Bosancianu, and Littvay 2019). Latent variables were random intercepts.

We used the flexible structural means modelling (SMM) method for comparing groups because it allows for high power due to correction for unreliability, and bypasses assumptions of zero measurement error, measurement invariance, lack of multicollinearity, homogeneity of variance, covariance, and regression (Breitsohl 2019). Moreover, the absence of a younger hearing-impaired group resulted in a fractional factorial design, complicating the disentanglement of a main effect of age from a main effect of hearing loss. The multilevel path analysis framework was used to handle the factorial design. Arousal data were analysed using the same approach; results of arousal ratings are displayed in Appendix B.

### Model fit

The models had a saturated covariance structure (variances and covariances among the repeated-measures outcomes were freely estimated) at the within- and between-persons levels. Neither the between-group variances nor the covariances were constrained, as the hypotheses focused on mean differences. Because the model was saturated, its fit is necessarily perfect. The model featured more parameters than clusters, raising concerns about the potential untrustworthiness of standard errors for specific parameters; however, exploratory analysis while constraining the variances and covariances did not sufficiently reduce the number of parameters to remedy this concern and considerably deteriorated model fit, so a saturated covariance structure was used.

## Experiment 1: results

### Main effects

The three groups had the same number and causal order of variables, suggesting that they are not different at a fundamental level, and allowing subsequent assessment of parameter equalities (Grace 2003). Wald tests of parameter constraints were used to test contrasts of mean differences for each level-1 and level-2 variable. The joint null hypothesis test for the main effect of group membership was not significant (*z* = 5.350, *p* = .069). Individual contrasts revealed that the younger listeners with normal hearing group reported significantly higher valence than the older listeners with hearing loss group (*z* = 15.124, *p* < .001). The older listeners with typical hearing group did not significantly differ from either the younger listeners with normal hearing or older listeners with hearing loss groups (*z* = 3.466, *p* = .063, and *z* = 2.832, *p* = .092, respectively). There was a significant main effect of dialogue-to-background ratios; participants reported higher valence in the +12 dB dialogue-to-background ratio conditions compared to +7 dB dialogue-to-background ratio conditions (*z* = 40.912, *p* < .001). Lastly, there was a significant main effect of speaker type (*z* = 17.666, *p* < .001). Participants reported higher valence in the stereo compared to television speaker conditions (*z* = 10.877, *p* = .001). They also reported significantly higher valence in the stereo condition compared to the surround sound condition (*z* = 15.031, *p* < .001). Moreover, there was no significant difference between the television speaker and surround sound conditions (*z* = 0.041, *p* = .840). However, the main effects should be interpreted with caution, due to the presence of significant interactions between predictor variables.

### Interactions

**Figure 2.**
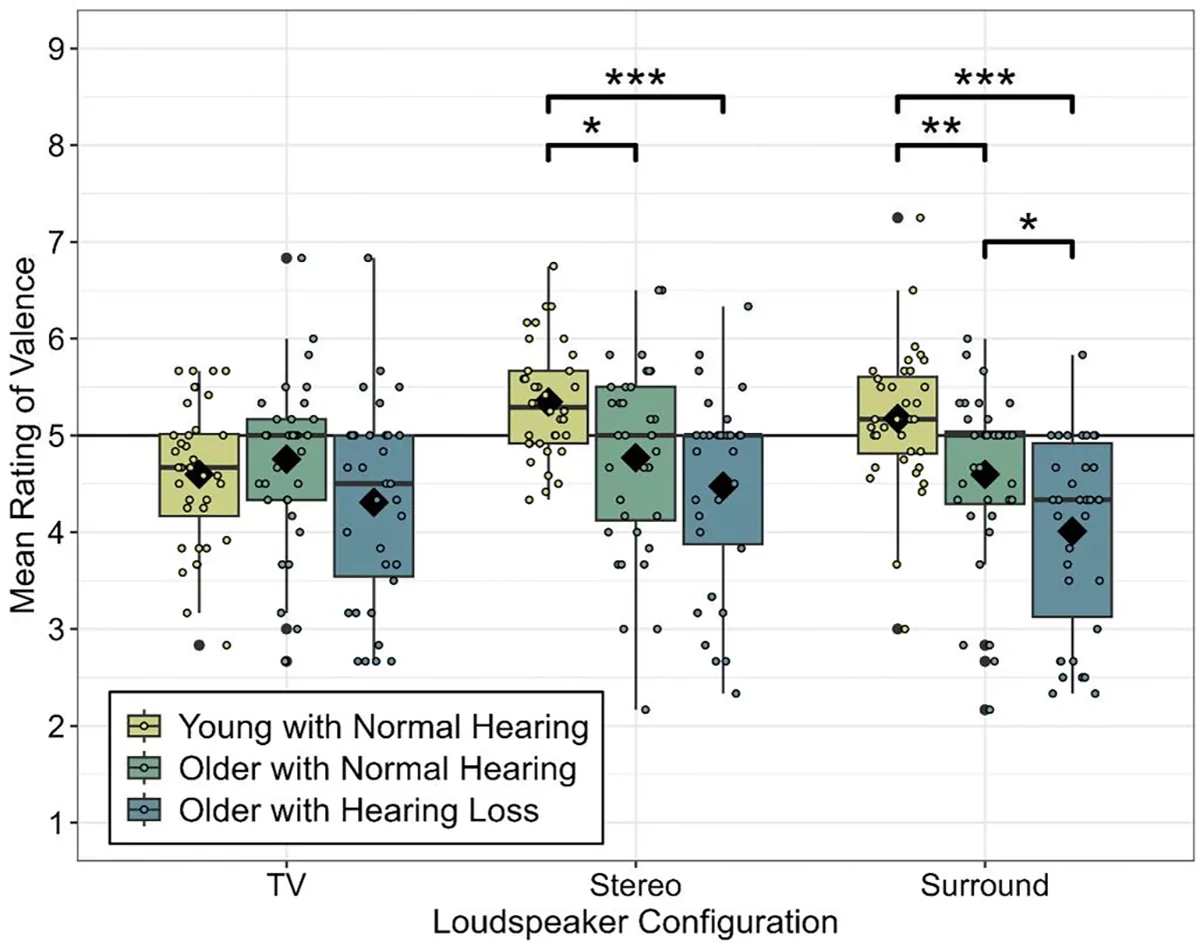
Mean ratings of valence for each participant group and each speaker configuration. Boxplots represent median valence and the 25^th^-75^th^ percentile. Diamonds indicate mean ratings. Pairwise comparisons were conducted separately within each group. *** indicates *p* <.001, ** indicates *p* < .01, and * indicates *p* <.05. (AUDIO-ONLY).

Analyses revealed no significant three-way interaction. However, there was a significant interaction between speaker configuration and group membership (see Figure 2). The difference between younger listeners with normal hearing and older listeners with typical hearing was larger in the stereo loudspeaker condition than television speaker (*z* = 9.008, *p* = .003), and mean difference in valence between older listeners with typical hearing and older listeners with hearing loss was greater in the surround sound loudspeaker condition than the stereo loudspeaker condition (*z* = 4.199, *p* = .041). The difference between younger listeners with normal hearing and older listeners with typical hearing was not significantly different in the stereo loudspeaker versus surround sound loudspeaker conditions (*z* = 0.059, *p* = .809)

Within the television loudspeaker condition, there was no difference in valence ratings between groups. Within the stereo speaker condition, there was no significant difference between older listeners with typical hearing and older listeners with hearing loss, but younger listeners with normal hearing reported significantly higher valence than both older listeners with typical hearing (*z* = 6.300, *p* = .012) and older listeners with hearing loss (*z* = 15.453, *p* < .001). Within the surround sound loudspeaker condition, younger listeners with normal hearing reported significantly higher valence ratings than older listeners with typical hearing (*z* = 6.944, *p* = .008), which in turn had significantly higher ratings than older listeners with hearing loss (*z* = 4.242, *p* = .040). Younger listeners with normal hearing had significantly higher valence ratings than older listeners with hearing loss (*z* = 23.401, *p* < .001). In summary, age was associated with lower valence ratings towards recorded media when the loudspeaker was surround sound; hearing loss was associated with reduced valence ratings only when the loudspeaker was surround sound.

**Figure 3.**
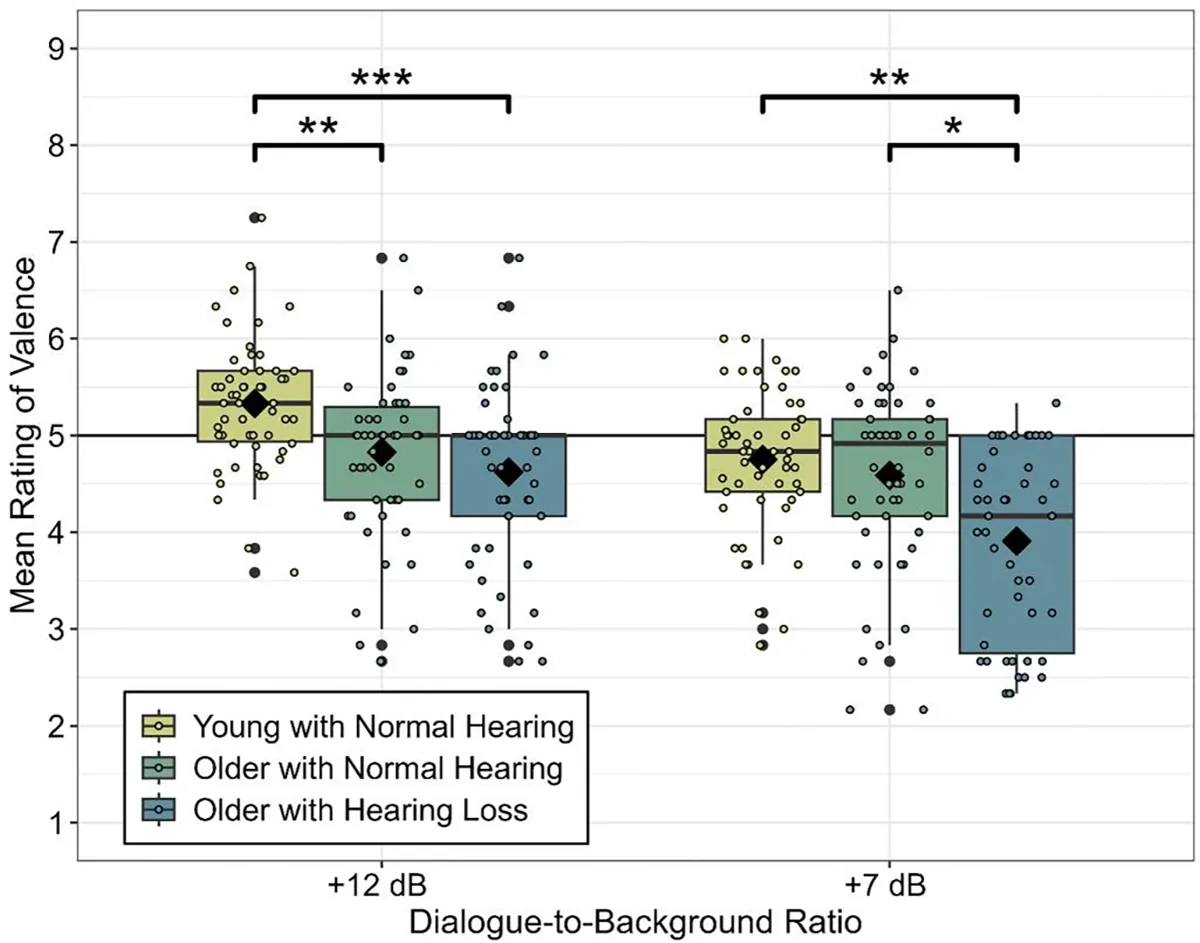
Mean ratings of valence for each dialogue-to-background ratio (DBR) and participant group. Boxplots represent median valence and the 25^th^-75^th^ percentile. Diamonds indicate mean ratings. Pairwise comparisons were conducted separately at each DBR. *** indicates *p* <.001, ** indicates *p* < .01, and * indicates *p* <.05. (AUDIO-ONLY).

There was also a significant interaction between group membership and dialogue-to-background ratios (see Figure 3). Within the +7 dB dialogue-to-background ratio, older listeners with hearing loss group had lower ratings of valence compared to the older listeners with typical hearing group (*z* = 6.573, *p* = .010). Within the +12 dB dialogue-to-background ratio condition, there was again no significant difference in valence ratings between older listeners with typical hearing and older listeners with hearing loss, but younger listeners with normal hearing ratings were significantly higher than both older listeners with typical hearing (*z* = 7.689, *p* = .006) and older listeners with hearing loss (*z* = 13.92, *p* < .001) ratings. Within the +7 dB dialogue-to-background ratio condition, there was no significant difference between younger listeners with normal hearing and older listeners with typical hearing valence ratings, but older listeners with hearing loss valence ratings were significantly lower than both older listeners with typical hearing (*z* = 5.124, *p* = .024) and younger listeners with normal hearing (*z* = 10.858, *p* = .001) ratings. In summary, age was related to lower valence ratings only when dialogue-to-background ratio was +12 dB, while hearing loss was associated with lower valence ratings only when dialogue-to-background ratio was +7 dB.

## Experiment 1: discussion

In Experiment 1, we evaluated ratings of valence in response to auditory-only recorded media for adults with and without hearing loss. We observed that group differences associated with age and hearing loss were conditional, dependent on both loudspeaker configuration and dialogue-to-background ratios.

### Hearing loss

Adults with hearing loss demonstrated lower ratings of valence, but only in the surround sound condition or when the dialogue-to-background ratio was +7 dB. These findings are partially consistent with previous work using either nonspeech sounds (Picou 2016; Picou et al. 2021) or speech sounds (Christensen et al. 2019). The finding that adults with hearing loss have different ratings of valence to sounds than their peers with typical hearing (both older and younger adults) in the current study is remarkable and complements findings with EMA reported previously (Holman and Naylor 2025).

The modifying effects of loudspeaker configuration were opposite to our expectations; we expected surround sound to increase ratings of valence. Given that spatial hearing abilities worsen with sensorineural hearing loss (Noble, Byrne, and Ter-Horst 1997), it could be that the surround sound configuration induced a soundscape that is too spatially complex. However, this hypothesis needs to be tested directly with psychophysics measures combined with surveys of valence in future works.

### Ageing

Compared to young adults with normal hearing, older adults provided lower ratings of valence in both stereo and surround sound conditions, and when the dialogue-to-background ratio was favourable (+12 dB). These results contrast with non-significant effects of age on ratings of valence shown previously with nonspeech sounds (Picou 2016) but are consistent with ageing-related vocal emotion recognition deficits (Christensen et al. 2019), likely consistent with our foreground stimuli being dialogues.

It is unclear why differences related to age were present only in the +12 dB dialogue-to-background ratio and only under the stereo or surround sound speaker configurations. Perhaps deficits in spatial hearing from ageing may only manifest in the presence of sufficiently audible and spatially complex soundscapes. This is consistent with ageing related deficits (independent of hearing loss) in spatial hearing (Eddins, Ozmeral, and Eddins 2018). Future work is required to test how one’s spatial hearing abilities affects valence perception in separated sound streams. Overall, the present findings could partially explain why older adults do not enjoy television watching as much as their peers with normal hearing.

Importantly, the above observations are specific to auditory-only signals and might not be representative of patients’ media watching experiences. We expect patients frequently listen to and also watch recorded media. Access to visual cues has been shown to enhance ratings of valence compared to ratings of auditory-only stimuli (Buono et al. 2021) and, in some cases, to counteract the effects of audio filtering (White, Hornsby, and Picou 2025). The purpose of Experiment 2 was to investigate the same questions as Experiment 1, but with audiovisual stimuli. It was expected that associations with hearing loss and valence would be less evident when visual cues were also present, but that relationships between age and valence ratings would persist even with visual cues present.

## Experiment 2: materials and methods

The same participants completed Experiment 2 as Experiment 1. Half of the participants completed testing in Experiment 1 before participating in Experiment 2. Different clips were included in Experiment 2 than in Experiment 1. The video components were presented from the same television that delivered the audio signals in Experiment 1 and the opaque black cloth that obscured visual cues on the screen was removed. Video materials were output via HDMI to a Sharp Model LC 32-LB481U Roku TV 5316X with software version 7.0.0. Otherwise, all equipment was identical to Experiment 1, as was the analysis strategy.

## Experiment 2: results

### Main effects

The joint null hypothesis test for the main effect of group membership was not significant. However, there was a significant main effect of dialogue-to-background ratio (*z* = 38.145, *p* < .001). Participants reported significantly higher valence in the +12 dB dialogue-to-background ratio conditions compared to +7 dB dialogue-to-background ratio conditions. There was a significant main effect of speaker configuration (*z* = 12.573, *p* = .002). Participants reported higher valence in the television loudspeaker compared to stereo conditions (*z* = 5.318, *p* = .021). They also reported significantly higher valence in the television loudspeaker configuration compared to the surround sound configuration (*z* = 11.298, *p* = .001). There was no significant difference between the stereo and surround sound configurations when collapsing across group membership. However, the main effects should be interpreted with caution due to the presence of significant interactions.

### Interactions

**Figure 4.**
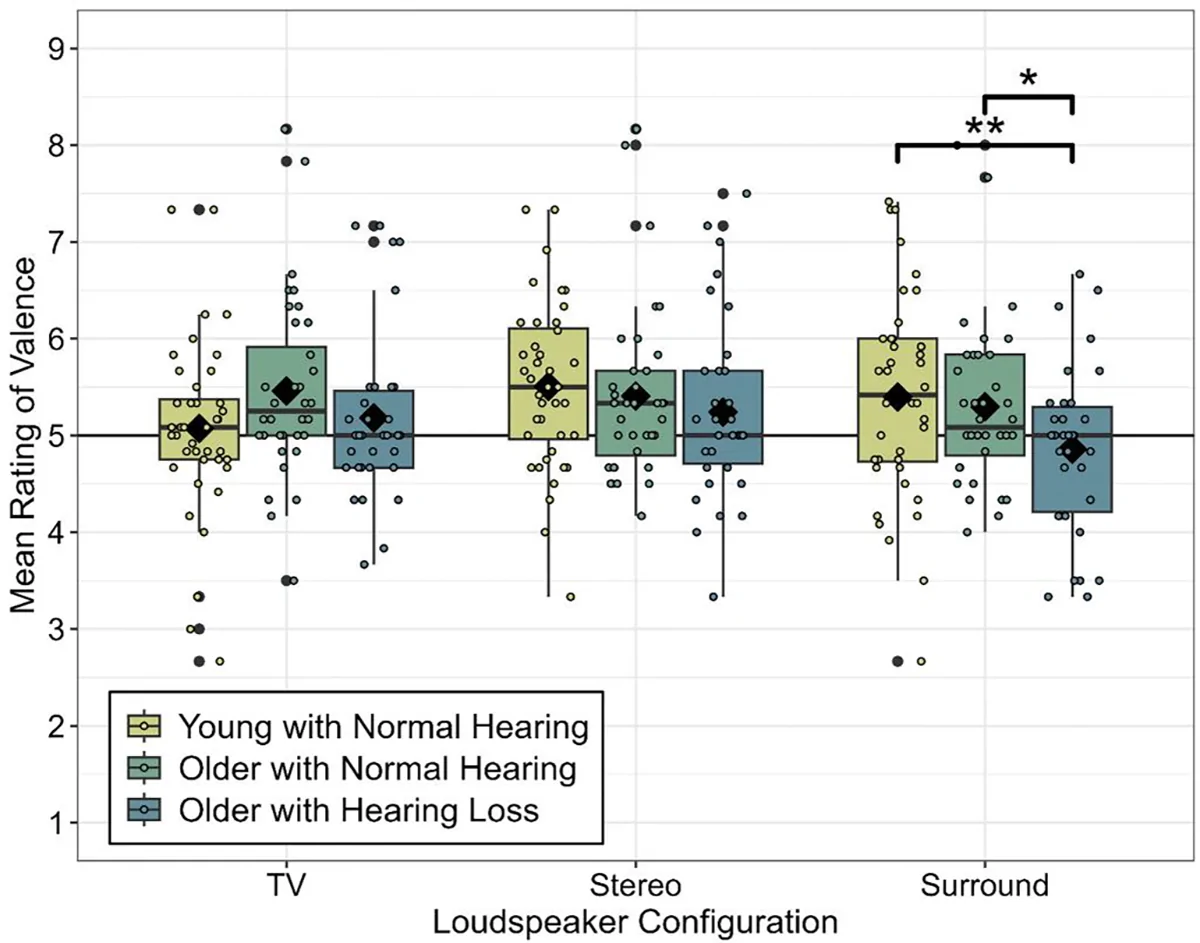
Mean ratings of valence for each participant group and each speaker configuration. Boxplots represent median valence and the 25^th^-75^th^ percentile. Diamonds indicate mean ratings. Pairwise comparisons were conducted separately within each group. ** indicates *p* < .01, and * indicates *p* <.05. (AUDIOVISUAL).

**Figure 5.**
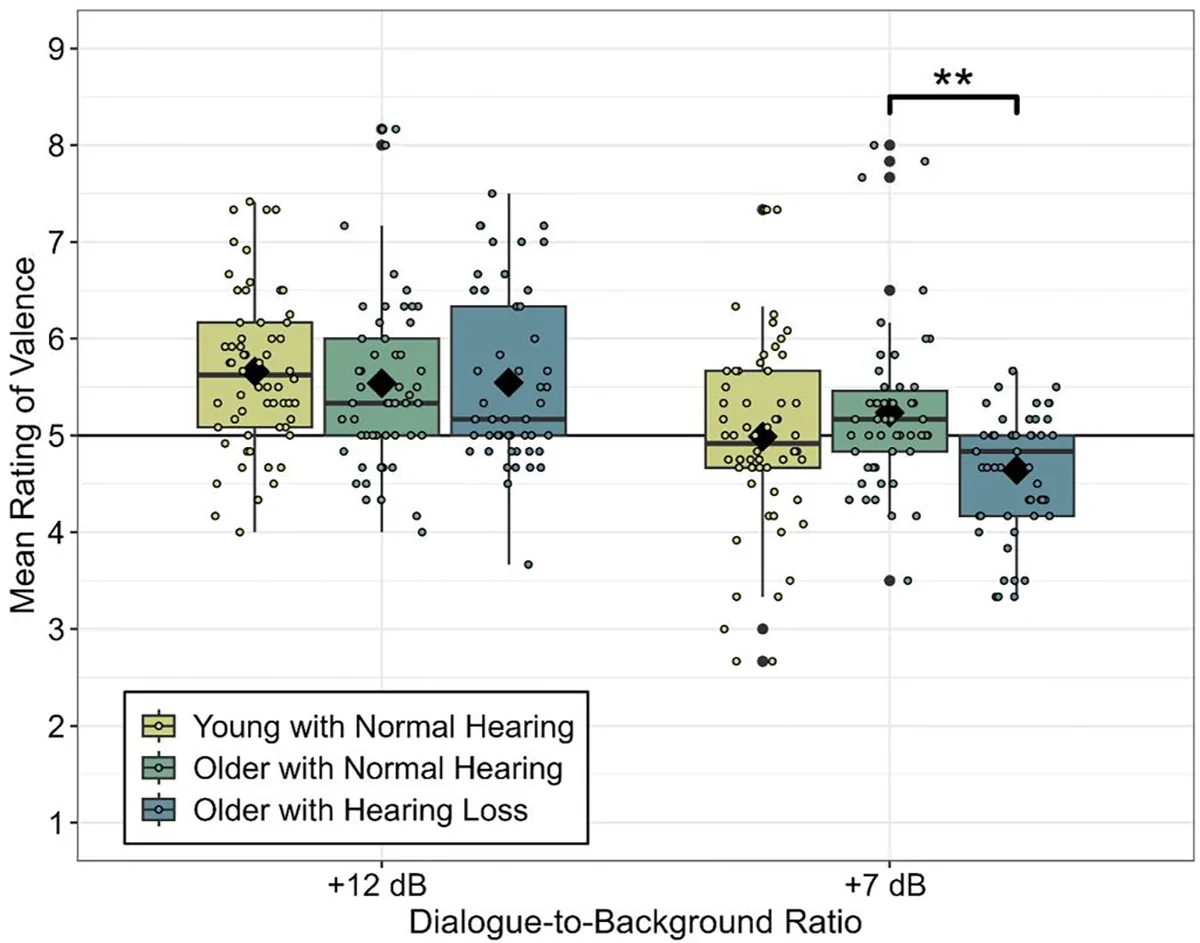
Mean ratings of valence for each dialogue-to-background ratio (DBR) and participant group. Boxplots represent median valence and the 25^th^-75^th^ percentile. Diamonds indicate mean ratings. Pairwise comparisons were conducted separately at each DBR. ** indicates *p* < .01. (AUDIOVISUAL).

There was no significant three-way interaction and no two-way interaction between dialogue-to-background ratio and loudspeaker configuration. However, there were significant interactions between loudspeaker configuration and group membership (see Figure 4) and between dialogue-to-background ratio and group membership (see Figure 5).

Regarding the interaction between loudspeaker configuration and group membership, mean differences of valence ratings between younger listeners with normal hearing and older listeners with typical hearing were smaller for television than for stereo sound loudspeaker configuration (*z* = 8.607, *p* = .003). Mean differences of valence ratings between younger listeners with normal hearing and older listeners with typical hearing were also smaller for surround than for stereo speaker configuration (*z* = 14.477, *p* < .001). Mean differences of valence ratings between older listeners with typical hearing and older listeners with hearing loss were smaller for television than for stereo speaker configuration (*z* = 4.826, *p* = .028). There were no significant mean differences of valence ratings between older listeners with typical hearing and older listeners with hearing loss for surround versus stereo speaker configurations.

Within both the television and stereo speaker configurations, there was no significant difference in valence ratings between groups. Within the surround sound configuration, the only significant contrast was that older listeners with hearing loss reported significantly lower valence than both older listeners with typical hearing (*z* = 4.620, *p* = .032) and younger listeners with normal hearing (*z* = 8.241, *p* = .004).

Regarding the group by dialogue-to-background ratio interaction, within the +12 dB condition, there was no difference in valence ratings between groups. Within the +7 dB dialogue-to-background ratio condition, the only significant contrast was that older listeners with hearing loss reported significantly lower valence than older listeners with typical hearing (*z* = 7.488, *p* = .006).

## Experiment 2: discussion

In Experiment 2, participants had access to both audio and visual modalities. Consistent with our findings with audio-only stimuli, the older listeners with hearing loss provided significantly lower valence ratings than their typical hearing peers, only when the surround sound loudspeakers were used or when the dialogue-to-background ratio was +7 dB. In contrast to Experiment 1, there was no significant difference in valence ratings between the two normal hearing groups across loudspeaker configurations. These findings demonstrate that hearing loss is associated with reduced valence even with visual cues present, but associations with age did not persist with the addition of visual cues.

The absence of associations with age in any loudspeaker configurations or dialogue-to-background ratio may suggest that visual cues mitigated for age-related deficits the stereo and surround sound configurations that were observed in Experiment 1 (auditory only). This finding was consistent with the literature that suggests minimal effects of ageing on emotional responses to stimuli, particularly when visual cues are available (e.g., Wieser et al. 2006).

It is notable that reduced valence ratings associated with hearing loss in the surround sound configuration and in the poorer dialogue-to-background ratio (+7 dB) could not be remedied by the addition of visual cues. Although visual modality has been shown to be dominant in emotion processing (e.g. Collignon et al. 2008), the concurrent degraded auditory input for adults with hearing loss had a significant modulating effect on the integrated emotion perception (Rosenfeld and Steffens 2019), a phenomenon we have also shown previously with non-speech sounds (Buono et al. 2021). Combined, these findings from both nonspeech sounds and recorded media support that hearing loss may affect emotional response to auditory-visual stimuli.

## General discussion

The extant literature largely focused on speech intelligibility or clarity for recorded media listening experience (Ricketts et al., 2026; Shirley 2013). The current work adds to this line of work with insights on listeners’ socioemotional experience while watching recorded media. For both audio-visual and audio-only stimuli, we found that older listeners with hearing loss provided significantly lower valence ratings than their typical hearing peers, but only when the surround sound loudspeaker configuration was used or when dialogue-to-background ratio was +7 dB. However, the association between age and ratings of valence was less consistent and depended on modality, dialogue-to-background ratio, and loudspeaker configuration.

Our findings in the surround sound condition were surprising, as we expected loudspeaker configurations aiming for envelopment or spaciousness would increase valence ratings (Moiragias and Mourjopoulos 2023). However, the observation of the negative effect of hearing loss itself is broadly in line with Holman and Naylor (2025). Without their hearing aids, adults with hearing loss reported lower valence across situation types, including television viewing, compared to the group with normal hearing. This finding lends consistency and empirical support for the ecological validity of the current study. We have also shown that similar negative impact of hearing loss observed with nonspeech sounds (Picou 2016) may be present when listening to recorded media. Combined, these studies highlight the negative impact of hearing loss on perceived valence, and the importance of considering the socioemotional well-being aspects of our patients.

The current data demonstrates benefits of favourable dialogue-to-background ratio for watching recorded media, with or without visual cues, especially for the older listeners with hearing loss. We have shown that improvement in dialogue-to-background ratio benefits rated valence, which aligns with a common reason for watching television – improving emotion and mood (Barwise, Bellman, and Beal 2020).

To this end, we have arrived at two relatively straightforward clinical implications for our older patients with hearing loss. First, based on our data, surround sound loudspeakers do not enhance ratings of valence for this group when watching dialogue-focused recorded media. In conjunction with our companion work showing reduced speech intelligibility with this loudspeaker configuration (Ricketts et al., 2026), we do not recommend the use of surround sound system for older listeners with hearing loss. Second, if the media of interest was recorded with object-based codecs (like Direct X), improvement in dialogue-to-background ratio are expected to better improve emotional response.

## Limitations and future directions

The current work has several limitations. Firstly, there was not a group of younger listeners with hearing loss. Thus, our interpretation of impact of hearing loss should be limited to older patients. Future studies should include younger adults with hearing loss to achieve a full factorial design for evaluating emotional responses to recorded media.

The second limitation relates to the selection of media content for our stimuli. Since preference for surround sound over stereo sound may depend on the type of media material (Kerins 2013), our findings with surround sound loudspeaker could potentially differ if the media genre we chose was different. One direction of future studies is to include recorded media that does not have a heavy focus on conversational dialogues.

Additionally, the stimuli used in the two experiments were from different portions of different shows. It was thus not possible to directly address effect of modality. However, the selection strategy was identical for both experiments, thus it would be unlikely that there were systematic effects of narrative differences between media clips.

Sound quality and intelligibility ratings were also collected as a part of a larger experiment and reported elsewhere (Ricketts et al., 2026). It is possible that valence ratings are partially driven by perceived sound quality and/or intelligibility, but rigorous investigation of such associations is beyond the scope of the current study. Future work will aim to mechanistically partition the variance in perceived valence explained by sound quality and intelligibility, above and beyond the experimental factors in the current study.

Lastly, although there were no asymmetries in hearing based on no difference in pure-tone average larger than 15 dB HL. There were 7 older listeners with typical hearing and 8 older listeners with hearing loss had asymmetrical hearing if defined by stricter criteria (10 dB difference at three frequencies or 15 dB difference at two frequencies). Slight hearing asymmetries may impact spatial hearing abilities; therefore, the group differences between younger listeners with normal hearing and the two older listener groups may be partially influenced by asymmetrical hearing.

In addition to spatial hearing, other factors should be included in future studies. For example, ratings of valence in response to recorded media might be affected by at-home TV watching preferences (e.g., loudspeaker set-up, group vs. individual watching), use of captioning for improved dialogue recognition, listener strategy (i.e., focus on semantics vs. raw acoustics) or use of assistive devices (e.g., cochlear implants/hearing aids). Each one of these factors has the potential to improve speech recognition, but also affect ratings of valence and should thus be carefully considered in the future.

## Conclusion

In the current study, we evaluated ratings of valence to auditory-only and auditory-visual recorded media for adults with and without hearing loss with listening situations that varied by loudspeaker configuration and dialogue-to-background ratio. Consistent across both audio-only and audio-visual experiments, adults with hearing loss provided lower ratings of valence in response to recorded media than did adults with normal or typical hearing, but only when a surround sound system was used or when the dialogue-to-background ratio was less favourable (+7 dB). These findings provide clinical implications for recommendations on loudspeaker configurations and desired dialogue-to-background ratio. Specifically, the findings support efforts to improve the dialogue-to-background ratios of television shows and movies, but do not support surround sound systems as an intervention to improve listening or viewing experiences. The study also presents a step towards using ecological stimuli in a laboratory setting to evaluate ratings of valence to sounds in situations with direct, real-world implications.

## Supplementary Material

Supp 1

Supp 2

Supplemental data for this article can be accessed online at https://doi.org/10.1080/14992027.2026.2710964.

## Data Availability

The dataset for this work is available upon request.

## References

[R1] Annerstedt M, Jönsson P, Wallergård M, Johansson G, Karlson B, Grahn P, Hansen AM, and Währborg P 2013. “Inducing Physiological Stress Recovery with Sounds of Nature in a Virtual Reality Forest–Results from a Pilot Study.” Physiology & Behavior 118:240–250. 10.1016/j.physbeh.2013.05.023. 23688947

[R2] Armstrong M 2016. From clean audio to object based broadcasting. BBC Research & Development White Paper, WHP324. London, United Kindom.

[R3] Barwise P, Bellman S, and Beal V 2020. “Why Do People Watch So much Television and Video? Implications for the Future of Viewing and Advertising.” Journal of Advertising Research 60 (2):121–134. 10.2501/JAR-2019-024.

[R4] Bradley MM, and Lang PJ 1994. “Measuring Emotion: The Self-Assessment Manikin and the Semantic Differential.” Journal of Behavior Therapy and Experimental Psychiatry 25 (1):49–59. 10.1016/0005-7916(94)90063-9. 7962581

[R5] Bradley MM, and Lang PJ 2007. “Emotion and motivation.” In Cacioppo JT, Tassinary LG, & Berntson GG (Eds.), Handbook of psychophysiology (3rd ed., 581–607. Cambridge University Press. Cambridge, United Kindom. 10.1017/cbo9780511546396.025.

[R6] Breitsohl H 2019. “Beyond ANOVA: An Introduction to Structural Equation Models for Experimental Designs.” Organizational Research Methods 22 (3):649–677. 10.1177/1094428118754988.

[R7] Buono GH, Crukley J, Hornsby BWY, and Picou EM 2021. “Loss of High- or Low-frequency Audibility Can Partially Explain Effects of Hearing Loss on Emotional Responses to Non-speech Sounds.” Hearing Research 401:108153. 10.1016/j.heares.2020.108153. 33360158

[R8] Christensen JA, Sis J, Kulkarni AM, and Chatterjee M 2019. “Effects of Age and Hearing Loss on the Recognition of Emotions in Speech.” Ear and Hearing 40 (5):1069–1083. 10.1097/AUD.0000000000000694. PMC6606405 30614835

[R9] Collignon O, Girard S, Gosselin F, Roy S, Saint-Amour D, Lassonde M, and Lepore F 2008. “Audio-visual Integration of Emotion Expression.” Brain Research 1242: 126–135. 10.1016/j.brainres.2008.04.023. 18495094

[R10] Davidson RJ, Sherer KR, and Goldsmith HH 2009. Handbook of affective sciences. Oxford, United Kindom: Oxford University Press.

[R11] Depp CA, Schkade DA, Thompson WK, and Jeste DV 2010. “Age, Affective Experience, and Television Use.” American Journal of Preventive Medicine 39 (2): 173–178. 10.1016/j.amepre.2010.03.020. PMC3593658 20621265

[R12] Dubno JR, Dirks DD, and Morgan DE 1984. “Effects of Age and Mild Hearing Loss on Speech Recognition in Noise.” The Journal of the Acoustical Society of America 76 (1):87–96. 10.1121/1.391011. 6747116

[R13] Eddins AC, Ozmeral EJ, and Eddins DA 2018. “How Aging Impacts the Encoding of Binaural Cues and the Perception of Auditory Space.” Hearing Research 369:79–89. 10.1016/j.heares.2018.05.001. PMC6196106 29759684

[R14] Fernández-Aguilar L, Ricarte J, Ros L, and Latorre JM 2018. “Emotional Differences in Young and Older Adults: Films as Mood Induction Procedure.” Frontiers in Psychology 9:1110. 10.3389/fpsyg.2018.01110. PMC6037940 30018584

[R15] Grace JB 2003. “11 Comparing Groups Using Structural Equations.” Structural Equation Modeling: applications in Ecological and Evolutionary Biology :281. Cambridge, United Kindom: Cambridge University Press.

[R16] Holman JA, and Naylor G 2025. “The Influence of Hearing Loss and Hearing Aid Use on Experienced Emotion in Everyday Listening Situations.” Clinical Rehabilitation 39 (6):770–783. 10.1177/02692155251326830. PMC12141770 40070164

[R17] Holman T 2012. Sound for film and television. Burlington, Massachusetts, USA: Routledge.

[R18] Huang H, Picou EM, and Martens WL 2026. “Relationship Between Spectrotemporal Modulation in Non-speech Sounds and Valence Ratings for Adults with and without Hearing Loss (L).” The Journal of the Acoustical Society of America 159 (4):3084–3087. 10.1121/10.0043329. 41949642

[R19] International Organization for Standardization 2017. Acoustics-Statistical distribution of hearing thresholds related to age and gender. International Organization for Standardization.

[R20] Kerins M 2013. Understanding the impact of surround sound in multimedia. Psychology of music in multimedia, Oxford University Press, New York, 365–390.

[R21] Kozloff S 2000. Overhearing film dialogue. University of Berkeley, California, USA: California Press.

[R22] Lang PJ 1995. “The Emotion Probe. Studies of Motivation and Attention.” The American Psychologist 50 (5):372–385. 10.1037//0003-066x.50.5.372. 7762889

[R23] Lin FR, Thorpe R, Gordon-Salant S, and Ferrucci L 2011. “Hearing Loss Prevalence and Risk Factors among Older Adults in the United States.” The Journals of Gerontology. Series A, Biological Sciences and Medical Sciences 66 (5):582–590. 10.1093/gerona/glr002. PMC3074958 21357188

[R24] Moiragias G, and Mourjopoulos J 2023. “A Listener Preference Model for Spatial Sound Reproduction, Incorporating Affective Response.” PLoS One 18 (6):e0285135. 10.1371/journal.pone.0285135. PMC10266670 37315025

[R25] Mulligan K, and Scherer KR 2012. “Toward A Working Definition of Emotion.” Emotion Review, 4 (4):345–357. 10.1177/1754073912445818.

[R26] Muthén LK, and Muthen B 2017. Mplus user’s guide: Statistical analysis with latent variables, user’s guide. Los Angeles, California, USA: Muthén & Muthén.

[R27] Noble W, Byrne D, and Ter-Horst K 1997. “Auditory Localization, Detection of Spatial Separateness, and Speech Hearing in Noise by Hearing Impaired Listeners.” The Journal of the Acoustical Society of America 102 (4):2343–2352. 10.1121/1.419618. 9348693

[R28] Pavic K, Vergilino-Perez D, Gricourt T, and Chaby L 2024. “Age-related Differences in Subjective and Physiological Emotion Evoked by Immersion in Natural and Social Virtual Environments.” Scientific Reports 14 (1):15320. 10.1038/s41598-024-66119-5. PMC11222553 38961132

[R29] Picou EM 2016. “How Hearing Loss and Age Affect Emotional Responses to Nonspeech Sounds.” Journal of Speech, Language, and Hearing Research: JSLHR 59 (5):1233–1246. 10.1044/2016_JSLHR-H-15-0231. 27768178

[R30] Picou EM, Rakita L, Buono GH, and Moore TM 2021. “Effects of Increasing the Overall Level or Fitting Hearing Aids on Emotional Responses to Sounds.” Trends in Hearing 25:23312165211049938. 10.1177/23312165211049938. PMC8825634 34866509

[R31] Richter ME, and Chatterjee M 2021. “Weighting of Prosodic and Lexical-Semantic Cues for Emotion Identification in Spectrally Degraded Speech and With Cochlear Implants.” Ear and Hearing 42 (6):1727–1740. 10.1097/AUD.0000000000001057. PMC8545870 34294630

[R32] Ricketts TA, Krivoshey R, Santos J, Huang H, and Em P 2026 “How Speaker Configuration of Recorded Media Affects Subjective Ratings for Listeners with and without Hearing Loss.” Journal of Speech, Language, and Hearing Research.1–14. 10.1044/2026_JSLHR-25-00127 PMC13475062 42335004

[R33] Rosenfeld N, and Steffens J 2019. “Effects of Audiovisual Congruency on Perceived Emotions in Film.” Psychomusicology: Music, Mind, and Brain 29 (4):200–208. 10.1037/pmu0000242.

[R34] Russell JA 1980. “A Circumplex Model of Affect.” Journal of Personality and Social Psychology 39 (6):1161–1178. 10.1037/h0077714.

[R35] Russell JA, and Mehrabian A 1977. “Evidence for a Three-factor Theory of Emotions.” Journal of Research in Personality 11 (3):273–294. 10.1016/0092-6566(77)90037-x.

[R36] Shirley BG 2013. Improving television sound for people with hearing impairments. University of Salford: United Kingdom.

[R37] Silva BC, Bosancianu CM, and Littvay L 2019. Multilevel structural equation modeling. Thousand Oaks, California, USA: Sage Publications.

[R38] Steiger JR 2005. “Audiologic Referral Criteria: Sample Clinic Guidelines.” The Hearing Journal 58 (5):38–39. 10.1097/01.HJ.0000287174.23086.36.

[R39] Strelcyk O, and Singh G 2018. “TV Listening and Hearing Aids.” PloS One 13 (6):e0200083. 10.1371/journal.pone.0200083. PMC6025866 29958299

[R40] White L, Hornsby B, and Picou E 2025. “Bandpass Filtering and Congruence Affect Emotional Responses to Auditory and Auditory–Visual Nonspeech Stimuli.” Perspectives of the ASHA Special Interest Groups 10 (6): 1767–1779. 10.1044/2025_PERSP-24-00184.

[R41] Wieser MJ, Mühlberger A, Kenntner-Mabiala R, and Pauli P 2006. “Is Emotion Processing Affected by Advancing Age? An Event-related Brain Potential Study.” Brain Research 1096 (1):138–147. 10.1016/j.brainres.2006.04.028. 16750819

[R42] Yang L, Cao C, Kantor ED, Nguyen LH, Zheng X, Park Y, Giovannucci EL, Matthews CE, Colditz GA, and Cao Y 2019. “Trends in Sedentary Behavior Among the US Population, 2001–2016.” JAMA 321 (16):1587–1597. 10.1001/jama.2019.3636. PMC6487546 31012934

