# Supplementary material for: Valence ratings of recorded media for adults with and without hearing loss
Source: Int J Audiol. Author manuscript; Available in PMC 2026 Sep 26. (PMC13615645; doi:10.1080/14992027.2026.2710964)
Supplement: Supplementary file 1 [file NIHMS2207195-supplement-Supp_1.docx]

Supplemental Information Appendix A

**Stimulus selection and manipulation**

Based on the fact that a listener’s experience, and thus preference, depends on a multitude of factors (e.g., material, background sound, emotional response) (Kerins, 2013; Moiragias & Mourjopoulos, 2023), our stimulus selection aimed to include a variety of materials. The current work selected materials with realism from commercial television shows and movies, to provide word to word variability in talker and background sound type. We screened approximately 100 television show and movie clips based on the procedures below. Our ultimate goal was to evaluate valence ratings for two different dialogue-to-background ratios and three different loudspeaker presentation configurations for a total of 6 listening conditions.

Criteria for inclusion as test material included: 1) continuous dialogue for at least 60 seconds during which the dialogue and non-dialogue (background) levels were relatively constant (based on subjective judgement), 2) the source material was available in 5.1 surround format and included audible (target) dialogue only in the center channel, 3) root-mean-square (rms) average dialogue levels in the center channel that were at least 14 dB higher than all other signals in the center channel, 4) inclusion of dialogue with a variety accents, 5) a range of background sound types including music, sound effects and background conversation, and 6) dialogue with male or female talkers with a variety of speaking rates. Use of a range of different materials was desirable to improve generalization of the results in part, because foreign dialects, noise in the listening environment, poor diction and high speech rates can also negatively affect dialogue recognition (Armstrong, 2016; Strelcyk & Singh, 2018).

After screening more than 100 clips for possible inclusion, a total of twelve, 60-second clips (6 audio and 6 audiovisual) were digitized to form the final test materials. For each stimulus set (audio, audiovisual), the 6 clips included: 1) British-accented male and female speech in background of classical music, 2) American-accented male speech in a background of modern music 3) American-accented male and female speech in a background of modern music, 4) American-accented female speech in a background of cafeteria noise, 5) a conversation among several American-accented talkers in a noisy restaurant, 6) a conversation among several British- and American accented talkers in background noise.

After the clips were selected and time edited to meet study criteria, the audio was modified via sound editing software (Adobe Audition, Version 2017.1.1, Build 10.1.1.11) to manipulate presentation level and dialogue-to-background ratio (DBR) *within* all clips while maintaining the natural sound characteristics as much as possible. Our DBR was derived by using our surround sound system. To prepare the signals for DBR measurement, we first band-pass filtered the signals (selected clips) from 353 to 5657 Hz to eliminate the frequency ranges for which there is little important speech information. This procedure resulted in a frequency range encompassing approximately 89% of important speech information (SII; ANSI S3.6, 1997). Then, we removed any silence in excess of 5 ms. Last, we compared the relative rms level of sound field recordings of the dialogue in the center channel to the non-dialogue level measured from the other four channels. The resulting rms level comparison is our DBR.

The test materials (unfiltered and previously unmodified) were then modified based on these level measurements. To ensure the loudness (LUFS re: ITU BS.1770) for the unaltered center channel (dialogue) of all clips was equivalent across clips, the level of all sound channels in each clip was adjusted in unison. Due to the focus on speech intelligibility and clarity in the companion study (Ricketts et al., Submitted) the low-frequency effects channel was then removed (the frequency range of these signals preclude a meaningful effect on dialogue audibility). The total level of the unfiltered non-dialogue sounds in the other four channels was then adjusted to manipulate DBR in 2-3 second blocks, while maintaining dialogue loudness. Within these blocks, DBRs were allowed to vary to preserve realism. Further, the exact duration of these blocks varied somewhat to avoid artificially manipulating dialogue-to-background ratio mid-word. +7- and +12-dB DBRs were the most frequently measured DBRs in the media clips screened for potential study inclusion, and therefore were chosen for the study.

References

Armstrong, M. (2016). From clean audio to object based broadcasting. *BBC Research & Development White Paper, WHP324*.

Bradley, M. M., & Lang, P. J. (1994). Measuring emotion: the self-assessment manikin and the semantic differential. *Journal of behavior therapy and experimental psychiatry*, *25*(1), 49-59.

Kerins, M. (2013). Understanding the impact of surround sound in multimedia. *Psychology of music in multimedia, Oxford University Press, New York*, 365-390.

Moiragias, G., & Mourjopoulos, J. (2023). A listener preference model for spatial sound reproduction, incorporating affective response. *PLoS One*, *18*(6), e0285135.

Ricketts, T. A., Krivoshey R., Santos, J., Huang, H., & EM, P. (Submitted). How speaker configuration of recorded media affects subjective ratings for listeners with and without hearing loss. *Journal of Speech, Language, and Hearing Research*.

Strelcyk, O., & Singh, G. (2018). TV listening and hearing aids. *PLoS One*, *13*(6), e0200083.
