# Supplementary material for: Valence ratings of recorded media for adults with and without hearing loss
Source: Int J Audiol. Author manuscript; Available in PMC 2026 Sep 26. (PMC13615645; doi:10.1080/14992027.2026.2710964)
Supplement: Supplementary file 2 [file NIHMS2207195-supplement-Supp_2.docx]

**Ratings of Arousal**

**Experiment 1 (Auditory only)**

***Results***

*Model fit*

The model has a saturated covariance structure at the within- and between-persons levels. Neither the between-group variances nor the covariances were constrained, as the hypotheses focused on mean differences. Because the model is saturated, its fit is necessarily perfect. The model featured more parameters than clusters, raising concerns about the potential untrustworthiness of standard errors for specific parameters; however, constraining the variances and covariances did not sufficiently reduce the number of parameters to remedy this concern and considerably deteriorated model fit, so a saturated covariance structure was used.

*Main effects, interactions*

The joint null hypothesis test for the main effect of group membership was significant (*z* = 7.842, *p* = 0.020). Individual contrasts revealed that the younger listeners with normal hearing reported significantly higher arousal than the older listeners with normal hearing (*z* = 4.132*, p = 0.0421*). However, the older listeners with normal hearing did not significantly differ from the older listeners with hearing loss (*z* = 0.111, *p* = 0.739).

Due to the limited variance of the response variance (arousal), no main effects of dialogue-to-background ratio or loudspeaker configurations were recorded, nor were any interactions.

**Experiment 2 (Audiovisual)**

***Results***

*Model fit*

The model has a saturated covariance structure at the within- and between-persons levels. Neither the between-group variances nor the covariances were constrained, as the hypotheses focused on mean differences. Because the model is saturated, its fit is necessarily perfect. The model featured more parameters than clusters, raising concerns about the potential untrustworthiness of standard errors for specific parameters; however, constraining the variances and covariances did not sufficiently reduce the number of parameters to remedy this concern and considerably deteriorated model fit, so a saturated covariance structure was used.

*Main effects, interactions*

The joint null hypothesis test for the main effect of group membership was significant (*z* = 6.265, *p* = 0.044). Individual contrasts revealed that the younger listeners with normal hearing reported significantly higher arousal than the older listeners with normal hearing (*z* = 5.265*, p* = 0.0212). However, the older listeners with normal hearing did not significantly differ from the older listeners with hearing loss (*z* = 0.417, *p* = 0.518).

Again, due to the limited variance of the response variance (arousal), no main effects of dialogue-to-background ratio or loudspeaker configuration were recorded, nor were any interactions.

**General discussion:**

Our analyses showed that for both experiments (i.e., with and without visual cues), younger listeners with normal hearing reported significantly higher arousal than the older listeners with normal hearing. In contrast, neither loudspeaker configuration nor dialogue-to-background ratio significantly affected ratings of arousal. These findings suggest that older age may be associated with decreased arousal when listening to recorded media. The nonsignificant association between hearing loss and arousal rating is consistent with our past works using nonspeech sounds (Picou, 2016; Picou et al., 2021) but contrasts a recent EMA study (Holman & Naylor, 2025). The discrepancies across studies may relate to differences in experimental design, as Holman and Naylor (2025) surveyed more listening situations than just recorded media watching as in the current work. The lack of significant effects of loudspeaker configuration and dialogue-to-background ratio suggest that ratings of arousal may not be sensitive to spatial separation of sound sources, or better audibility of running dialogues.

**References**

Holman, J. A., & Naylor, G. (2025). The influence of hearing loss and hearing aid use on experienced emotion in everyday listening situations. *Clinical Rehabilitation*, *39*(6), 770-783.

Picou, E. M. (2016). How hearing loss and age affect emotional responses to nonspeech sounds. *Journal of Speech, Language, and Hearing Research*, *59*(5), 1233 - 1246. <https://doi.org/https://doi.org/10.1044/2016_JSLHR-H-15-0231>

Picou, E. M., Rakita, L., Buono, G. H., & Moore, T. M. (2021). Effects of increasing the overall level or fitting hearing aids on emotional responses to sounds. *Trends in hearing*, *25*, 1-13.
